# Supplementary material for: MetaRibo-Seq measures translation in microbiomes
Source: Nat Commun. 2020 Jun 29;11:3268. doi: 10.1038/s41467-020-17081-z (PMC7324362; doi:10.1038/s41467-020-17081-z)
Supplement: Supplementary file 10 — Supplementary Data 7 [file 41467_2020_17081_MOESM10_ESM.zip › File2/Confidence_High_Taxonomy/217789_out.krona.html]

Javascript must be enabled to view this page.

members
magnitude
magnitudeUnassigned
count
unassigned
taxon
rank

217789\_out

3

3
superkingdom
2

phylum
3
976

3
class
200643

171549
order
3

2
family
815

2
genus
816

2
species

SRS147039\_contig\_number\_3977SRS147377\_contig\_number\_1372
821

171552
1
family

genus
1
838

species
1

SRS147151\_contig\_number\_14585
28137
